# Evaluation of the Stability, Revision Rate, and Complication Profile of Combined Anterior Cruciate Ligament Reconstruction with Lateral Extra-Articular Tenodesis and Hughston Procedure in Anterior Cruciate Ligament and Medial Collateral Ligament Injury: An 8-Year Cohort Study

**DOI:** 10.1177/23259671241309651

**Published:** 2025-03-03

**Authors:** Gian Andrea Lucidi, Emanuele Altovino, Stefano Di Paolo, Piero Agostinone, Francesca Maria Marziano, Nicola Pizza, Giacomo Dal Fabbro, Alberto Grassi, Stefano Zaffagnini

**Affiliations:** † Clinica II, IRCCS, Istituto Ortopedico Rizzoli, Via Pupilli, Bologna, Italy; ‡ Department of Biomedical and Neuromotor Sciences, Anatomy Centre, University of Bologna, Via Irnerio Bologna, Italy; Investigation performed at Clinica II, IRCCS, Istituto Ortopedico Rizzoli Istituto di Ricovero e Cura a Carattere Scientifico, Via Pupilli, Bologna, Italy

**Keywords:** knee, ligament, ACL, MCL, anteromedial instability, anterolateral instability, pivot shift

## Abstract

**Background:**

Anterior cruciate ligament (ACL) tears combined with medial collateral ligament (MCL) injury has been associated with an increased rate of ACL reconstruction (ACLR) failure, high-grade pivot shift (PS), and lower return to sports rate. On the other hand, medial-sided procedures in the setting of ACLR are associated with knee stiffness and arthrofibrosis.

**Purpose/Hypothesis:**

This study aimed to compare clinical scores, objective knee laxity, failure, and complication rates in 2 different patient groups. The combination of ACL reconstruction with LET and the Hughston procedure yields comparable failure rates, complication rates, and clinical outcomes to ACL reconstruction with LET in patients without medial instability.

**Study Design:**

Cohort study; Level of evidence, 3.

**Methods:**

A group of patients had a combined ACL and MCL injury grade 2 with chronic instability and underwent ACLR associated with lateral extra-articular tenodesis (LET) and the Hughston procedure (Hughston group). The control group included matched patients with isolated ACL lesion without medial instability who underwent ACL reconstruction with LET (control group). Patient-reported outcome measures, complications, and reoperations were collected for both groups. A clinical evaluation was performed including objective anteroposterior laxity measurement (KT-1000 arthrometer) and PS quantification. The primary outcomes were ACL revision and ACL-clinical failure, a composite parameter of anteroposterior and rotatory laxity. A test for 2-way analysis of variance for repeated measures was performed to assess the between-group differences (*P* < .05). Surgical and clinical failure were assessed via Kaplan-Meier method.

**Results:**

A total of 70 patients (35 per group) were enrolled in the present study at a follow-up of 8.1 ± 2.7 years. All the patient-reported outcome measures significantly improved at the final follow-up with no difference between the 2 groups (*P* > .05). ACL revision was performed in 2 of 35 (5.7%) patients in both groups (*P* = .79). A total of 10 patients (4 in the Hughston group and 6 in the control group) were excluded from the analysis of the clinical failures due to contralateral-side injury. Clinical failure was identified in 7 of 28 (25.0%) patients in the Hughston group and 5 of 29 (17.2%) in the control group (*P* = .59). Reoperation due to knee stiffness was required only in 1 of 35 patients (2.9%) of the Hughston group.

**Conclusion:**

Due to its simplicity and cost-effectiveness, the Hughston technique should be included in the orthopaedic surgeon's armamentarium for the treatment of moderate anteromedial instability in combined ACL and MCL injury. Moreover, the outcomes and failure rate of the Hughston technique combined with an ACLR + LET are similar to that of an ACLR + LET used to treat an isolated ACL injury.

The association of anterior cruciate ligament (ACL) and medial collateral ligament (MCL) tears is a common clinical scenario and accounts for almost 20% of knee ligamentous injuries.^46^ Furthermore, a recent study has found that in almost two-thirds of the so-called “isolated” ACL tears, there are some MCL injuries that are overlooked.^55^

Additionally, recent evidence demonstrated that an involvement of the medial compartment during the mechanism of ACL injury entails a more severe grade of soft tissue and bony structural damage potentially leading to an increased knee rotatory instability.^1,15,45^

While a registry study has shown the importance of surgical management of medial-sided injury in reducing the ACL reconstruction (ACLR) failure rate, the same study also reported significantly lower clinical scores and lower sports participation when an MCL reconstruction was performed in this combined setting.^49^ However, since medial-sided instability represents a broad spectrum, not all patients may require an invasive procedure such as a formal MCL reconstruction. In the past, alternative medial-side surgeries aimed at retensioning slackened structures have been proposed with satisfactory long-term clinical results.^25^ These procedures have been recently rediscovered and could be a promising surgical alternative treatment for patients with chronic mild valgus instability, but clinical studies are missing.^40^

Moreover, in past years, many authors have focused on the kinematic and clinical effect of the anterolateral instability (ALRI) on the outcomes of ACLR, and combined procedures including lateral extra-articular tenodesis (LET) have been shown to be effective in controlling rotatory instability and to reduce the failure rate.^13,19,47,48^ Even after decades of advancement, the imaging evaluation of injuries to the medial and posteromedial corner is still challenging, and the anteromedial and anterolateral rotatory instability remain mainly a clinical diagnosis without a consensus in surgical management.^5,21,32,37,40,41,45^

Moreover, in the setting of combined instability, the decision to associate both LET and medial-side knee procedures with the intra-articular ACLR could be questioned considering the possible higher rate of arthrofibrosis and knee complications historically associated with these procedures.^39,42^

Therefore, the aim of the present study was to evaluate clinical outcomes, failure rate, complications, and objective knee stability of a group of patients treated in the chronic setting with ACLR associated with LET and the Hughston procedure. We hypothesized similar clinical outcomes and failures but an increased rate of complications when compared with a control group of ACL-injured patients without MCL injury treated with ACLR and LET. A list of abbreviations used throughout this manuscript is detailed in Table 1.

**Table 1:** Abbreviations and Their Meaning

Abbreviation	Meaning
ACL	Anterior cruciate ligament
ACLR	Anterior cruciate ligament reconstruction
AMRI	Anteromedial instability
ALRI	Anterolateral instability
KiRA	Kinematic Rapid Assessment
KOOS	Knee Injury and Osteoarthritis Outcome Score
LET	Lateral extra-articular tenodesis
MCL	Medial collateral ligament
dMCL	Deep Medial Collateral Ligament
sMCL	Superficial Medial Collateral Ligament
MRI	Magnetic Resonance Imaging
POL	Posterior Oblique Ligament
PS	Pivot shift
ROM	Range Of Movement
RTS	Return to Sport

## Methods

### Ethics

The study was approved by the institutional review board of Istituto Ortopedico Rizzoli, Bologna (Prot gen CE AVEC 147/2021), and each patient gave informed consent.

### Patient Selection

A retrospective analysis was performed on patients who underwent arthroscopic ACLR + LET and medial capsular reefing using the Hughston technique between 2008 and 2019 at a single institution (Istituto Ortopedico Rizzoli di Bologna). A total of 35 patients (Hughston group) with a minimum follow-up of 3 years were included in the present study. Details regarding inclusion and exclusion criteria are summarized in Table 2.

**Table 2:** Inclusion and Exclusion Criteria^*a*^

Inclusion Criteria
Age between 15 and 60 years Minimum follow-up of 3 years ACL tears with high grade pivot shift (2+ or 3+) Patients who underwent primary ACL reconstruction + LET (control group) Patients with chronic, proximal MCL injury (>4 weeks) (Hughston group) Patients who underwent primary ACL reconstruction + LET combined with Hughston technique (Hughston group)
Exclusion Criteria
Patients with a history of previous knee surgery Patients with open physis Patients with other concomitant ligaments injury (PCL, LCL, PLC) Concomitant surgical procedures other than meniscal treatment Patients who underwent isolated Hughston procedures Patients with no endpoint at valgus stress requiring MCL reconstruction Patients with acute MCL injury (<4 weeks) Patients with distal MCL injury (“Stener-like” lesions) Patients who underwent ACL revision Patients with 1+ or 3+ valgus laxity

a ACL, anterior cruciate ligament; LCL, lateral collateral ligament; LET, lateral extra-articular tenodesis; MCL, medial collateral ligament; PCL, posterior cruciate ligament; PLC, posterolateral corner.

Patients of the Hughston group were matched 1:1 by age (±5 years), follow-up time (±3 years), sex (exact), and meniscal lesion with 35 patients with an isolated ACL injury that underwent ACLR with LET and no other additional procedures (control group).

Diagnosis of ACL and MCL tears was based on clinical and radiological (magnetic resonance imaging [MRI]) findings. ALRI was evaluated with anterior drawer, Lachman, and pivot-shift tests. Anteromedial instability (AMRI) was investigated with anterior drawer test with an externally rotated tibia and valgus test at 0° and 30°.

In the case of medial-sided injury, patients were first treated with 2 weeks of full extension brace and partial weightbearing with crutches; the brace was unlocked, and active and passive flexion-extension of the knee (0°-90°) was encouraged after 2 weeks. The brace was removed after 6 weeks.

At the time of surgery, patients underwent clinical examination under anesthesia to assess the grade of the anterolateral and medial-sided laxity. For patients with isolated ACL tears, the indications for primary ACLR + LET included positive Lachman combined with high-grade pivot shift (PS) (2+ or 3+). Those patients were included in the control group.

In the presence of medial-sided injury, the treatment of the medial structure was performed according to the intraoperative degree of medial instability after ACL graft and LET fixation. Indications for the Hughston procedure included patients with AMRI and valgus laxity (2+ with an endpoint at 30°) due to proximal damage of the medial structures. Patients with 3+ laxity and no endpoint at valgus stress, combined with ACL complete lesion, underwent ACLR + LET and MCL reconstruction. In the case of grade 1+ laxity, no treatment of the medial structures was performed.

**Figure 1.**
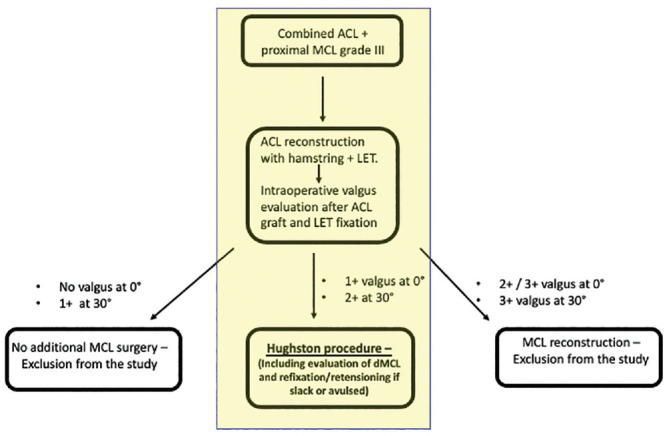
Flowchart for the surgical management of medial-sided injuries. ACL, anterior cruciate ligament; dMCL, deep MCL; LET, lateral extra-articular tenodesis; MCL, medial collateral ligament.

Patients with 3+ laxity or those with only 1+ laxity were excluded from the study. The intraoperative flowchart for clinical examination and surgical management of medial-sided injuries is summarized in Figure 1.

### Surgical Technique

After a standard clinical examination under anesthesia, a diagnostic arthroscopy was performed through standard anterolateral and anteromedial arthroscopic portals. The ACL remnant was then debrided, and meniscal procedures were performed as needed with either an all-inside suture or partial meniscectomy.

In all the patients, ACLR was performed with hamstring tendon autograft using the “over the top” technique with a LET.^17^

Briefly, the gracilis and semitendinous tendons were harvested proximally using an open tendon stripper while leaving the tibial insertion intact. The 2 tendons were harvested together to obtain a double-strand construct. The graft was then passed into the tibial tunnel, to reach the over-the-top position of the lateral femoral condyle where it was secured with 2 metal staples (8 mm) with the knee at 90° of knee flexion. The remaining part of the graft was passed below the iliotibial band but superficial to the lateral collateral ligament and pulled distally to the tibia, where it was fixed at the level of the Gerdy tubercle with a metal staple (6 mm) at 60° of knee flexion with the foot in neutral rotation.

After the ACLR and LET fixation, residual medial instability was investigated, performing an anteromedial drawer test and valgus test. In the case of AMRI and 2+ valgus laxity with an endpoint and MRI evidence of proximal damage to the medial structures, the Hughston procedure was performed.^26,40^

A longitudinal incision from the medial epicondyle to the medial joint line was performed. After skin and subcutaneous dissection, the sartorial fascia was incised exposing the underlying structure. In all cases, the superficial MCL (sMCL) fibers appeared to be slackened at their femoral end. With the knee flexed at 90°, a vertical incision was performed between the posterior border of the sMCL and the posterior oblique ligament (POL). At this moment, it was possible to evaluate the deep MCL (dMCL), the posteromedial capsule, and the POL. Once assessed and documented, the repair and/or retensioning of damaged or slackened structures was performed from posterior to anterior and deep to superficial. In a few cases, the inspection of the dMCL revealed an avulsion of the femoral portion, and a reattachment with anchors was performed as well.

After the repair of the deep structures, 4 No. 2 Ethibond (Ethicon; Johnson & Johnson) horizontal mattress sutures, at 1 cm apart each, were passed through the POL, the posteromedial capsule, and finally to the posterior border of the sMCL in a pants-over-vest fashion. A Kocher instrument was used to keep the POL advanced while the sutures were tied with the knee at 90° of flexion and slight varus stress and with the foot in neutral rotation. Last, layer 1 was closed using absorbable No. 1-0 Vicryl suture (Ethicon; Johnson & Johnson). The stability of the knee was confirmed both in extension and at 30° of flexion before the skin and subcutaneous tissue closure.

**Figure 2.**
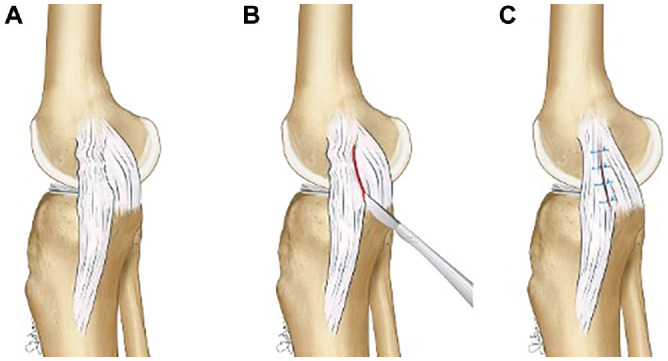
Illustration demonstrating (A) the slackened fibers of the superficial medial collateral ligament (sMCL) at their femoral portion anteriorly to the posterior oblique ligament (POL). (B) A vertical incision between sMCL and POL is then performed to evaluate the deep structures. (C) Horizontal mattress sutures, at 1 cm apart each, passed through the POL, the posteromedial capsule, and finally to the posterior border of the sMCL in a pants-over-vest fashion.

**Figure 3.**
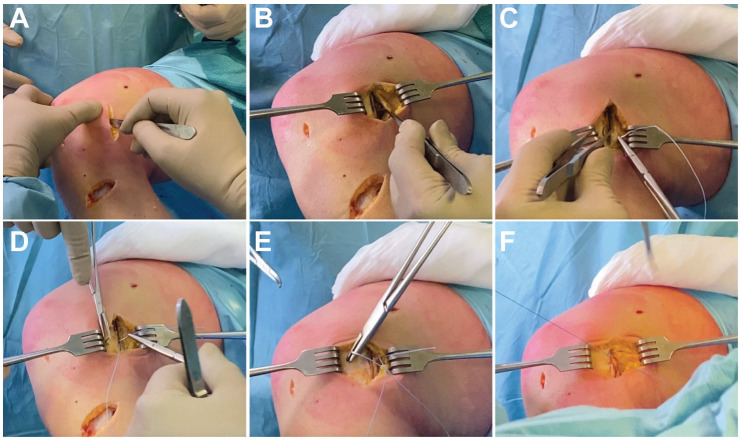
Intraoperative pictures of Hughston technique. (A) Longitudinal incision from the medial epicondyle to the medial joint line. (B) After incision of the sartorial fascia, with the knee flexed at 90°, a vertical incision is performed between the posterior border of the superficial medial collateral ligament (sMCL) and the posterior oblique ligament (POL). (C-E) Horizontal mattress sutures, at 1 cm apart each, passed through the POL, the posteromedial capsule, and finally to the posterior border of the sMCL in a pants-over-vest fashion. (F) At the end of the procedure, the proximal portion of the MCL appears to be retensioned.

The surgical technique of the Hughston procedure is illustrated in Figures 2 and 3.

### Rehabilitation Protocol

The limb was immobilized with a knee brace in full extension for 1 week in the Hughston group. After this period, the brace was unlocked, allowing passive and active knee flexion and extension from 0° to 90° for the first 3 weeks, with a progressive increase starting from the fourth week. After 3 weeks, the brace was used only to protect the knee during walking and it was removed after 6 weeks.

Partial weightbearing was allowed for the first 2 weeks with a progression to full weightbearing at the end of the fourth week. Stationary cycling and swimming were introduced 4 weeks after surgery.

The rehabilitation of the control group was similar. However, no brace was required, and the patients were allowed to bear weight as tolerated from the first week after surgery.

### Clinical Evaluation

At final follow-up, clinical evaluation was performed using Lachman and pivot-shift tests, range of motion (ROM) assessment, and valgus stress test at 0° and 30°. The objective anterior laxity was assessed using a KT-1000 arthrometer^14^ at 25 N and manual maximum; rotational laxity was investigated by performing a PS and quantified with a Kinematic Rapid Assessment (KiRA) accelerometer.^30^

Subjective clinical evaluation was performed using Lysholm, Knee injury and Osteoarthritis Outcome Score (KOOS), and Tegner score presurgery, as well as at the final follow-up in both groups. Reoperation and complications for every patient were collected as well.

Surgical failure was considered in every case of ACL graft tear (confirmed by either MRI or arthroscopy) or in case of ACL revision. Clinical failure was evaluated with a composite parameter of anteroposterior and rotatory laxity defined as ≥1 of the following objective criteria: moderate or severe PS (clinical grade ≥2 or KiRA acceleration ≥1.5 mm/s^2^ side-to-side difference), abnormal Lachman test (KT-1000 arthrometer side-to-side difference ≥5 mm) or graft rupture (confirmed by either MRI or arthroscopy).^14,34^ Medial instability was investigated with a manual valgus laxity test with the knee at 0° and 30°, compared with the contralateral limb.

### Statistical Analysis

The continuous parametric variables were expressed as mean ± SD, noncontinuous variables were presented as median and interquartile range, and categorical variables were expressed as numbers and percentages. Test for 2-way analysis of variance for repeated measures was performed to assess the between-group differences of continuous variables. The Student *t* test was used to compare each group with another in multiple comparison post hoc setting with Bonferroni correction. The Cohen *d* effect size and the mean difference between the groups (with 95% CI) were reported alongside the *P* value. Effect size was considered trivial, small, medium, and large for Cohen *d* values of <0.2, 0.2, 0.5, and 0.8, respectively. Differences were considered significant with *P* < .05. Chi-square test was used to compare frequencies between groups.

Survival analyses were performed using the Kaplan-Meier method, and surgical failure and clinical failure were used as separate endpoints for the survival analysis. Survival proportions at 2 years, 5 years, and final follow-up with standard errors were calculated. Hazard ratios with 95% CIs were also reported. All the statistical analysis were performed in MATLAB (The MathWorks).

## Results

**Figure 4.**
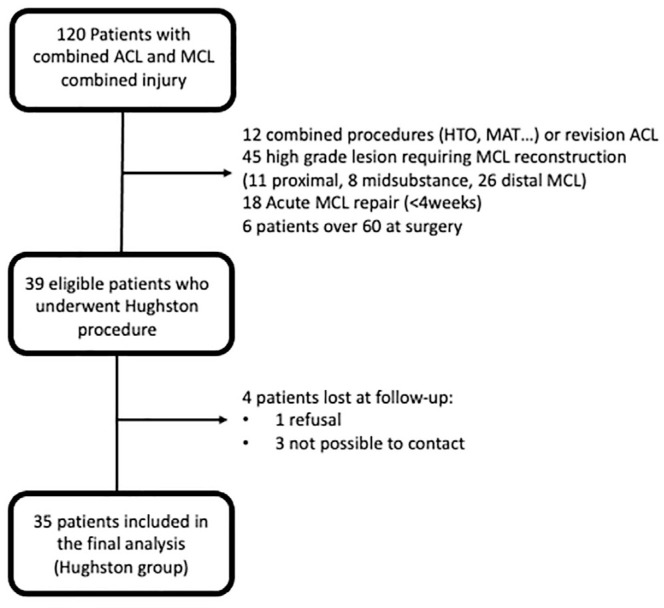
CONSORT (Consolidated Standards of Reporting Trials) flow diagram. ACL, anterior cruciate ligament; HTO, high tibial osteotomy; MAT, meniscal allograft transplantation; MCL, medial collateral ligament.

A total of 39 patients who had undergone a primary ACLR with LET and concomitant MCL injury treated with the Hughston technique met the inclusion criteria (Figure 4). Four patients were lost to follow-up, leaving 35 (90%) patients who were included in the Hughston group and were matched with 35 controls who had undergone a primary ACLR + LET for isolated ACL injury. In the Hughston group, 1 patient underwent dMCL reattachment due to chronic avulsion, and another 7 patients underwent dMCL retensioning using suture anchors.

The mean follow-up was 8.1 ± 2.7 years. There were no differences in demographic characteristics between the 2 groups (Table 3).

**Table 3:** Demographic Characteristics^*a*^

Measure	Hughston Group	Control Group	*P*
Sex, female/male	8/27	8/27	ns
Age, y	31.6 (13.2)	31.2 (12.9)	ns
Diabetes, yes/no	1/34	0/35	ns
Smoker, yes/no	7/28	6/29	ns
Trauma, D/I	4/31	1/34	ns
Meniscal lesion, yes/no	19/16	16/19	ns
Meniscal lesion, M/L	9/13	12/10	ns
Meniscal treatment, M/S	6/13	4/12	ns
Cartilage lesion, yes/no	2/33	0/35	ns
Time from injury to surgery, mo	5.1 (4.7)	6.5 (5.8)	ns

a Data are presented as n or mean (SD). D/I, direct/indirect; M/L, medial/lateral; M/S, meniscectomy/suture; ns, nonsignificant (*P* > .05).

All the patient-reported outcome measures significantly improved at the final follow-up. Reoperation was required in 5 of 35 (14.3%) patients in the Hughston group and in 4 of 35 (11.4%) in the control group (*P* = .72). There were 2 surgical failures (ACL revision) in both groups. The full list of reoperations is presented in Table 4. There was only 1 (2.9%) reoperation due to knee stiffness in the Hughston group, which required a manipulation under anesthesia.

**Table 4:** Reoperation Causes^*a*^

Surgery	Hughston Group	Control Group
Meniscal scaffold	1	0
Hardware removal	1	2
MUA	1	0
ACL revision	2	2
Total	5	4

a Data are presented as n.ACL, anterior cruciate ligament; MUA, mobilization under anesthesia.

### Subjective Evaluation

Contralateral-side ACL injury was reported in 4 of 35 (11.4%) and 6 of 35 (17.1%) patients of the Hughston and control groups, respectively (*P* = .50).

At the final follow-up, no statistically significant differences emerged between the clinical scores of the 2 groups (*P* > .05) (Table 5).

**Table 5:** Patient-Reported Outcome Measures^*a*^

Measure	Hughston Group	Control Group	*P*	Cohen *d* [95% CI]
VASr	1.5 (2.2)	0.6 (1.5)	.08	0.32 [–0.04 to 0.67]
VASs	2.5 (3.1)	1.5 (2)	.15	0.26 [–0.1 to 0.61]
Lysholm	92.4 (11)	95.7 (6.2)	.15	0.26 [–0.09 to 0.61]
KOOS ADL	95.1 (10.6)	97.8 (6.2)	.21	0.23 [–0.13 to 0.58]
KOOS Pain	92.7 (12.8)	94.9 (9.9)	.50	0.12 [–0.23 to 0.47]
KOOS QoL	77.6 (24.4)	79.6 (17.4)	.80	0.04 [–0.3 to 0.39]
KOOS Sport/Rec	80.8 (25.3)	90.6 (12.2)	.06	0.35 [–0.02 to 0.7]
KOOS Symptoms	89.8 (11.2)	88.3 (12.1)	.58	0.1 [–0.25 to 0.45]
Tegner FFU	5.2 (2.1)	4.9 (1.3)	.50	0.12 [–0.23 to 0.47]
Tegner postsurgery	5.8 (2.3)	5.7 (1.6)	.89	0.03 [–0.32 to 0.37]
Tegner preinjury	6.7 (2.2)	6.5 (1.4)	.66	0.08 [–0.27 to 0.42]
Tegner presurgery	2.4 (1)	2.9 (1)	.07	0.34 [–0.02 to 0.69]

a Data are presented as mean (SD) unless otherwise indicated. ADL, Activities of Daily Living; FFU, final follow-up; KOOS, Knee injury and Osteoarthritis Score; QoL, Quality of Life; Sport/Rec, Sport and Recreation; VAS, visual analog scale; VASr, VAS at rest; VASs, VAS in sport.

### Objective Evaluation

At final follow-up, no significant difference in pivot-shift acceleration, anteroposterior laxity, and ROM emerged between the 2 groups (Table 6).

**Table 6:** Objective Evaluation^*a*^

Measure	Hughston Group	Control Group	*P*	Cohen *d* [95% CI]
KiRA inj, mm/s^2^	3.2 (0.9)	3.1 (1.2)	.71	0.07 [–0.3 to 0.43]
KiRA contra, mm/s^2^	2.6 (0.8)	2.9 (1.6)	.62	0.11 [–0.33 to 0.55]
KiRA S-S, mm/s^2^	0.6 (0.9)	0.2 (1.2)	.90	0.03 [–0.41 to 0.47]
KT 25 N inj, mm	6.6 (2.6)	7.2 (2.8)	.15	0.28 [–0.1 to 0.64]
KT 25 N contra, mm	5.0 (1.7)	5.7 (2.3)	.15	0.34 [–0.12 to 0.79]
KT 25 N S-S, mm	1.7 (2.0)	1.5 (2.4)	.86	0.04 [–0.4 to 0.48]
KT man max inj, mm	9.3 (3.1)	8.8 (2.9)	.89	0.03 [–0.34 to 0.39]
KT man max contra, mm	7.2 (1.9)	7.4 (2.1)	.69	0.09 [–0.35 to 0.53]
KT man max S-S, mm	2.3 (2.8)	1.5 (2.2)	.73	0.08 [–0.36 to 0.52]
ROM active inj, deg	127.1 (7.1)	130.2 (5.2)	.10	0.31 [–0.06 to 0.69]
ROM passive inj, deg	131.9 (5.2)	130.9 (5.1)	.41	0.16 [–0.21 to 0.52]

a Data are presented as mean (SD) unless otherwise indicated. contra, contralateral limb; inj, injured; KiRA, Kinematic Rapid Assessment; KT 25 N, KT-1000 at 25 N; KT man max, KT-1000 at manual maximal force; ROM, range of motion; S-S, side-to-side difference.

### Clinical and Surgical Failure

**Figure 5.**
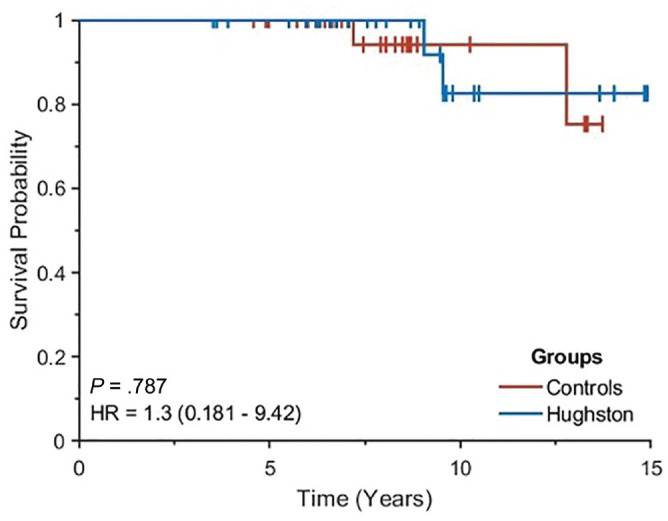
Kaplan-Meier curves describing the survivorship of the Hughston group (blue) and control group (red). HR, hazard ratio.

Surgical failure was identified in 2 of 35 (5.7%) occurring in the Hughston group and 2 of 35 (5.7%) occurring in the control group. No statistical difference emerged between the 2 groups (Figure 5).

Three patients in the Hughston group were interviewed by telephone and could therefore not be tested objectively.

A total of 10 patients (4 in the Hughston group and 6 in the control group) were excluded from the analysis of the clinical failures due to contralateral-side injury. Clinical failure was identified in 7 of 28 (25.0%) occurring in the Hughston group and 5 of 29 (17.2%) in the control group. No differences in survivorship from clinical failure were found between the Hughston group and the control group (*P* = .47). Survivorship from clinical failure is presented in Figure 5.

At the last follow-up, in the Hughston group, side-to-side differences in valgus opening at 30° of knee flexion was graded as 1+ in 4 patients (12%) and 2+ in 2 patients (6%).

## Discussion

The most important finding of the present study is that the presence of ACL and MCL tears with ALRI, the combination of ACLR + LET, and the Hughston procedure could be considered a valuable surgical strategy with satisfactory clinical results and low failure rate, comparable with ACLR + LET for isolated ALRI. Moreover, this procedure has been shown to be safe, without an increase in reoperation and complication rate.

There has recently been increased interest in medial-sided knee injuries, primarily because of the complex anatomy of the medial structure and the uncertainty of the outcomes in a combined setting.^12,22,31^ Several in vitro cutting studies have highlighted the biomechanical role of the dMCL, sMCL, and POL in resisting different loads applied to the knee joint.^6,7,8,20^ Similarly, many studies have focused on other grafts and configurations of medial-sided reconstruction.^44,53,56^ In the present study, patients underwent a surgical procedure aiming to retension the POL rather than “become or take the place of the ligament.”^38^

If left untreated, medial-sided injuries can increase knee rotatory instability and are associated with worse clinical outcomes and increased revision rate.^46^ A study found that concomitant sMCL lesion of grade 2 was associated with a 13-fold increase in the revision risk after ACLR.^2^ An increased ACL failure rate with conservatively treated MCL lesions has also been reported in registry studies^49^ and ACL revision cases.^4^ In the present study, the Hughston procedure associated with ACLR reduced the revision rate to results comparable with ACLR for an isolated ACL tear (6% in both groups). Moreover, no difference in clinical failure was found even after applying stricter failure criteria based on objective anteroposterior and rotatory laxity measurements.

While few studies have evaluated the Hughston procedure in primary ACL settings, some authors have investigated the short-term outcomes of combined ACL and MCL reconstruction with contradictory findings. In a short-term follow-up study investigating patients with ACL and MCL grade 2 with a “floating meniscus” sign, the revision rate was significantly lower (3% vs 29%) when the ACLR was combined with MCL reconstruction.^12^ On the other hand, in a randomized controlled trial of patients with ACL and MCL grade 3, no difference in subjective and objective outcomes was reported between conservative treatment and MCL reconstruction.^22^ A long-term follow-up study investigating the outcomes of combined ACL and grade 2 MCL tears reported good clinical scores and low failure rates in 57 patients treated with isolate double-bundle ACLR and nonoperative treatment of the MCL. Although this study suggests that conservative treatment of the MCL could represent an appropriate strategy, it should be underlined that the side-to-side difference reported with the Telos device (METAX Kupplungs und Dichtungs technik) under valgus stress was 1.7 mm.^58^

Interestingly, a recent study found that patients with persistent medial instability in the setting of ACL revision experience a lower failure rate if an MCL reconstruction rather than a medial capsular plication is performed in association with ACL revision.^3^ However, it is well-established that in a revision setting there is increased overall laxity compared with primary ACLR and that the outcome after ACL revision is related to multiple factors, including previous meniscal treatment, tibial slope, tunnel position, and cartilage status.^9,36^ Therefore, results of primary and revision ACL cases should not be pooled together.

Biomechanical studies have shown that untreated medial-sided injuries can result in increased anterior and rotational translation and that residual medial laxity puts the ACL graft under greater load, increasing the likelihood of graft failure.^6,33^

Other studies have found that there is a critical shared loading response among the superficial MCL and the POL.^20,43^ Therefore, a retensioning of these 2 structures together may determine a stabilizing effect against both valgus and internal-external rotation forces.^21^

Moreover, the proximal portion of the sMCL, unlike the distal portion, had an increased amount of soft tissue adherences that could tend to naturally disperse the load.^27^ This observation that the proximal sMCL portion is attached primarily to soft tissue rather than anchored directly to bone represents the biomechanical rationale for a soft tissue retensioning in chronic injuries of the POL over the proximal MCL.^27^

In the present study, an open approach with systematic evaluation of the dMCL allowed for identifying and treating these neglected injuries in 8 (23%) of the patients of the Hughston group. A recent study highlighted the incidence and the importance of dMCL tears in the setting of ACL and medial-sided injuries.^55^ The literature still debates whether the sMCL or the dMCL is the primary restraint to external rotation in the ACL-deficient knee. However, recent evidence suggests that both structures play a near-equal role in restraining AMRI. Therefore, a systematic approach and repair of all the damaged structures should be crucial since there is significant load sharing between these 2 structures.^7,52^ Moreover, after damage to the dMCL, an increase of 46% of the load on the ACL was found in recent biomechanical testing^7^; therefore, dMCL injuries should never be neglected.

On the other hand, biomechanical studies have shown that LET performed in combination with ACLR can offload the ACL graft up to 80%,^35,47^ and it is unknown if LET could be effective in reducing the failure rate in the presence of MCL deficiency, even if these factors work independently.^11^ Furthermore, even though LET is becoming extremely popular and is performed regularly in some categories of patients, there is a paucity of studies investigating the outcomes of combined ACLR with LET and the medial-sided procedure.

Although not significant, there was a trend (*P* = .06) toward less sports activity in the Hughston group compared with isolated ACLR, with an overall difference of 10 points in the KOOS Sport and Recreation subscale. Interestingly, recent studies from the Swedish National Register^49,50^ reported a lower return to sport at 1 year and worse subjective score (–14 points in the KOOS Sport and Recreation subscale) for patients requiring surgical treatment of the MCL.

With that said, concerns still exist regarding the use of LET because previous authors have reported overconstraint of the knee, arthrofibrosis, and a greater rate of complication after lateral procedures.^10,51,57^ Similarly, the most common complication after MCL surgery is postoperative arthrofibrosis.^28,29^ However, the present study showed that a LET is not associated with an increased risk of complication or reintervention for knee stiffness, even in the setting of combined ACLR and surgical MCL treatment if a supervised rehabilitation program focused on regaining knee ROM is started a few days after surgery.

The safety of LET has been investigated in a series of >500 patients by the SANTI group.^51^ Their results showed only a 4% of reoperation for stiffness-related complications, which is a percentage in line with that reported in the Multicenter Orthopaedic Outcomes Network cohort after isolated ACLR.^24^ Furthermore, data from the STABILITY trial have shown that LET may result in a slight increase of pain in the early postoperative period but at the final follow-up of 2 years it is not correlated with decreased range of motion, medical or surgical events, and overall reoperation rates.^23^

At the last follow-up, while severe medial instability was noted only in 2 cases (6%), a minor asymptomatic valgus opening was noted in additional 4 patients (12%). These results are in line with recent biomechanical studies investigating the effect of different medial reconstruction procedures on knee kinematics.^8,38,54^ All these studies showed that the medial stability could be significantly improved after a medial reconstruction but there is still no surgical strategy that can reproduce the intact state.^38,54^ Therefore, a moderate medial gapping at the last follow-up could be tolerated, especially considering that the surgical technique performed was a capsular plication aimed to restore the natural soft tissue tension rather than completely reconstructing the medial-sided anatomy.

Finally, we would like to underline that the present research aims not to advise for a chronic MCL treatment but rather to report the outcomes of a soft tissue retensioning technique that should become part of the surgical armamentarium of the sports medicine surgeon due to its simplicity and efficacy.

### Limitations and Strengths

The present study has several limitations. First, the study is designed as a retrospective case-control analysis. However, a strict matching process was performed to reduce risk of bias when evaluating the patients. The 2 groups of patients were similar in terms of demographics, meniscal lesions, and follow-up. Second, because of the rarity of chronic medial instability associated with ACL tears, it was only possible to include a limited number of patients and the possibility of type 2 error exists for some outcomes. Third, medial-sided stability was evaluated with a clinical examination and no objective quantification of the valgus laxity reduction was performed. Nevertheless, because of the costs and the reduced availability of quantification devices (eg, Telos), the evaluation of the valgus test has often been proposed in many other clinical studies as a reliable outcome.^4,12,46^ Moreover, a recent systematic review has shown that the diagnostic accuracy of MCL instability of stress radiography is similar to the clinical examination if the latter is performed under anesthesia.^37^ An additional limitation is related to the study design. Since almost all patients with moderate valgus laxity underwent the Hughston procedure, it was not possible to identify a control group with untreated medial instability. Therefore, the control group included patients with a different injury pattern (isolated ACL injury) who underwent ACLR + LET. While this approach compares patient-reported outcome measures, failures, and complications between these 2 groups, there is no direct comparison with patients with a similar injury pattern. Additionally, since the decision to perform the Hughston procedure was based on the intraoperative valgus laxity, it was impossible to provide data regarding how many patients did not require this additional medial procedure after the bracing treatment. Last is the lack of a systematic imaging evaluation at the last follow-up. However, the osteoarthritis progression in the 2 groups was not the study's main research question. Moreover, systematic reviews have shown that the MRI can reliably diagnose MCL lesions. However, it is not reliable in predicting the grade of MCL laxity^37^ and, therefore, it was performed only in case of reported knee instability to investigate potential ACL graft failure.

This research has several strengths that are important to highlight. This study was the first to investigate the outcomes of the Hughston technique performed in a contemporary setting using an arthroscopic technique and with a standard modern rehabilitation. Similarly, to the best of our knowledge, it is the first study to report on the outcomes of combined ACLR + LET and medial-sided procedure. Moreover, the length of the study was appropriate considering that the vast majority of ACLR failure occurs in the first few postoperative years.^16,18^ Finally, to not underestimate the failure rate, a careful instrumented clinical examination was performed on anteroposterior or rotational instability.

## Conclusion

Due to its simplicity and cost-effectiveness, the Hughston technique should be included in the orthopaedic surgeon's armamentarium for the treatment of chronic moderate AMRI in combined ACL and MCL injury. Moreover, the outcomes and failure rate of the Hughston technique combined with an ACLR + LET are similar to that of an ACLR + LET used to treat an isolated ACL injury.
